# The Study of Environment on Aboriginal Resilience and Child Health (SEARCH): study protocol

**DOI:** 10.1186/1471-2458-10-287

**Published:** 2010-05-28

**Authors:** The SEARCH Investigators

**Affiliations:** 1 The SEARCH Investigators, The Sax Institute PO Box 123, Broadway NSW 2007, Australia

## Abstract

**Background:**

Aboriginal Australians have a life expectancy more than ten years less than that of non-Aboriginal Australians, reflecting their disproportionate burden of both communicable and non-communicable disease throughout the lifespan. Little is known about the health and health trajectories of Aboriginal children and, although the majority of Aboriginal people live in urban areas, data are particularly sparse in relation to children living in urban areas.

**Methods/Design:**

The Study of Environment on Aboriginal Resilience and Child Health (SEARCH) is a cohort study of Aboriginal children aged 0-17 years, from urban and large regional centers in New South Wales, Australia. SEARCH focuses on Aboriginal community identified health priorities of: injury; otitis media; vaccine-preventable conditions; mental health problems; developmental delay; obesity; and risk factors for chronic disease. Parents/caregivers and their children are invited to participate in SEARCH at the time of presentation to one of the four participating Aboriginal Community Controlled Health Organisations at Mount Druitt, Campbelltown, Wagga Wagga and Newcastle. Questionnaire data are obtained from parents/caregivers and children, along with signed permission for follow-up through repeat data collection and data linkage. All children have their height, weight, waist circumference and blood pressure measured and complete audiometry, otoscopy/pneumatic otoscopy and tympanometry. Children aged 1-7 years have speech and language assessed and their parents/caregivers complete the Parental Evaluation of Developmental Status. The Study aims to recruit 1700 children by the end of 2010 and to secure resources for long term follow up. From November 2008 to March 2010, 1010 children had joined the study. From those 446 children with complete data entry, participating children ranged in age from 2 weeks to 17 years old, with 144 aged 0-3, 147 aged 4-7, 75 aged 8-10 and 79 aged 11-17. 55% were male and 45% female.

**Discussion:**

SEARCH is built on strong community partnerships, under Aboriginal leadership, and addresses community priorities relating to a number of under-researched areas. SEARCH will provide a unique long-term resource to investigate the causes and trajectories of health and illness in urban Aboriginal children and to identify potential targets for interventions to improve health.

## Background

Aboriginal Australians have a life expectancy more than ten years less than that of non-Aboriginal Australians [1], reflecting their disproportionate burden of both communicable and non-communicable disease. This disadvantage is apparent throughout the lifespan. Infections [2], injury [3] and social and emotional wellbeing problems [4] are all more prevalent amongst Aboriginal than non-Aboriginal children and mortality rates are significantly higher across all age groups [2]. While the disparity between Aboriginal and non-Aboriginal child health is undisputed, research to date has tended to be cross sectional, aimed primarily at particular health problems and disproportionately focused on the health status of children living in rural and remote communities. Consequently, Aboriginal child health is poorly described across a number of areas, few interventions have been rigorously tested [5], and little is known about the health of the 76% of Aboriginal people who live in urban areas [6]. This lack of evidence has serious implications for the development of effective policies and programs to improve Aboriginal child health.

Historically, Aboriginal health research has often been conducted on, rather than with, Aboriginal people with little benefit for the communities involved [7,8]. This legacy has resulted in reluctance by Aboriginal communities to participate in research, and the need for a rethinking of the basis for research in Aboriginal Health. A key element of current and future research is the forming of genuine partnerships between Aboriginal Communities and research organisations to allow research to be carried out that addresses community priorities, enables the informed and ethical engagement of community members in the research process, and facilitates the translation of research findings into improvements in health outcomes [9,10]. In order to progress the conduct of such research in New South Wales, the Coalition for Research to Improve Aboriginal Health was established in 2004 as a partnership between the Aboriginal Health and Medical Research Council of New South Wales (the peak body and voice of Aboriginal communities on Aboriginal health matters in New South Wales) and the Sax Institute (an organisation dedicated to building partnerships between researchers and health policy and service delivery agencies to improve health).

One of the first activities undertaken by the Coalition for Research to Improve Aboriginal Health was consultations with members of the Aboriginal Health and Medical Research Council to determine the research priorities of the New South Wales Aboriginal community. These discussions identified strong preferences for research that: examined the health and wellbeing of children and families; looked at health holistically - taking into account housing, financial pressures and mental health; followed families for up to 20 years to see how their health progressed, and the things that predicted health getting better or worse; and included interventions in key areas - ear health and housing were nominated as particularly important.

The Study of Environment on Aboriginal Resilience and Child Health (SEARCH), the first major research program established by the Coalition, was designed to address these priorities. SEARCH is conducted in urban and large regional centers in New South Wales, the state with the largest Aboriginal population, due to an urgent need for data in this area. The Study is conducted in partnership with Aboriginal Community Controlled Health Organisations (ACCHOs), services which provide comprehensive primary health care to Aboriginal people in a culturally appropriate manner. It draws upon the expertise of leaders in Aboriginal health with that of researchers to conduct SEARCH in a culturally appropriate manner and the employment and training of Aboriginal researchers is prioritised.

The aim of SEARCH is to describe and investigate the causes of health and illness in approximately 1700 urban Aboriginal children aged 0-17 years, from around 700 families, with a focus on healthy environments and selected child health problems. This will be done initially through a cross sectional study of 1700 children living in urban and large regional centres and then continued via a prospective cohort study of these children over 5 years. Greater numbers of children will be recruited and followed over time, should funding allow. The child health problems prioritised by SEARCH are: injury; otitis media; vaccine-preventable conditions; mental health problems; developmental delay; obesity and; risk factors for chronic disease.

This paper describes the design and methods of SEARCH and provides preliminary information on participant characteristics.

## Methods

### Study population

**Figure 1:**
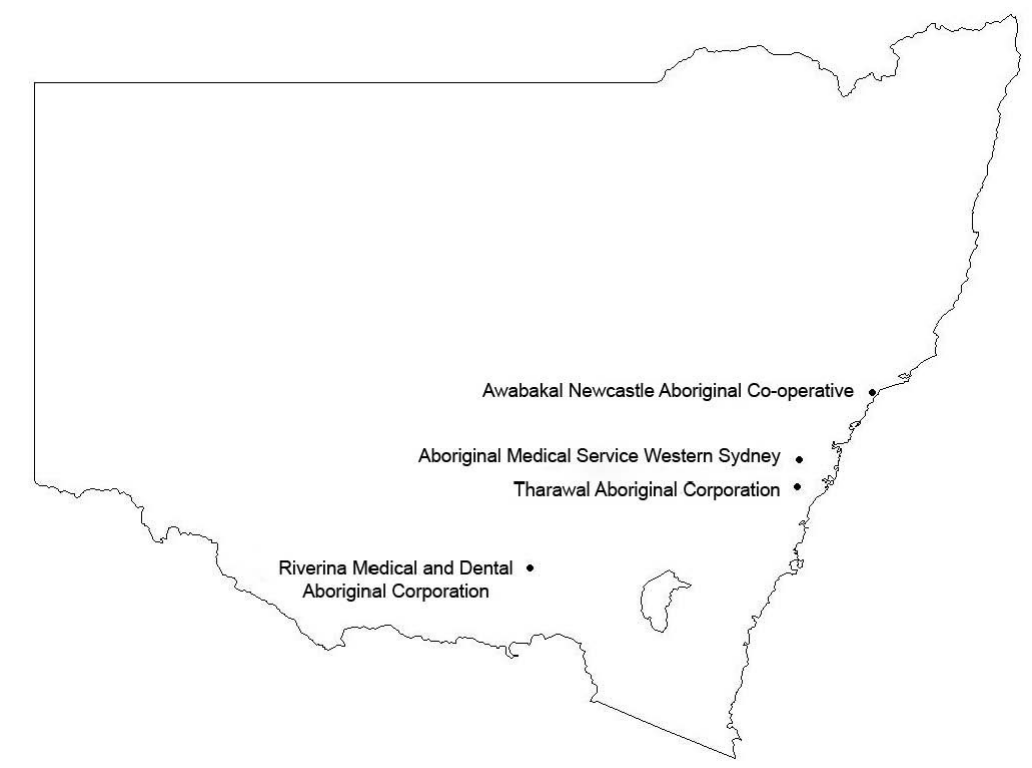
**Location of SEARCH recruitment centres**.

SEARCH is conducted in partnership with four ACCHOs, all of which are located in urban and large regional centres in NSW (Figure 1): Mount Druitt (Aboriginal Medical Service Western Sydney); Campbelltown (Tharawal Aboriginal Corporation); Wagga Wagga (Riverina Medical and Dental Aboriginal Corporation), and Newcastle (Awabakal Newcastle Aboriginal Co-operative). Local Aboriginal data collectors have been recruited and all data collection from participants occurs on site.

The study is aimed at Aboriginal children aged 0-17 years who attend a participating ACCHO, and their parent/caregivers. Children in the target population are eligible to participate provided their parent/caregivers were aged 16 years or over and are willing to provide contact information for follow up interviews.

Prior to recruitment commencing, the study is publicised at each ACCHO via community events, posters and leaflets. There are three main methods by which families are recruited into the study: 1) parents/caregivers are approached by a data collector in the waiting room of a participating ACCHO and invited to participate in the study; 2) parents/caregivers are informed about the study by their general practitioner (GP) or a health worker when attending an appointment at the ACCHO; and 3) in sites where it was deemed appropriate by the ACCHO, invitation letters are mailed out to families who use the service.

Families meeting inclusion criteria are invited to participate in SEARCH at the time of presentation at a participating ACCHO. Parents/caregivers are provided with a Participant Information Sheet by a data collector and talked through its contents. Willing parents/caregivers then sign a written consent form to participate in the study and permit future follow-up through additional data collection and data linkage, on behalf of themselves and their children. Adolescents aged 12-17 are given their own Participant Information Sheet and also sign their own consent form.

The study was approved by the ethics committees of the Aboriginal Health and Medical Research Council of New South Wales and of the University of Sydney.

### Governance

The SEARCH Steering Group is composed of the Study Investigators, representatives of all of the participating ACCHOs and the Aboriginal Health and Medical Research Council. The SEARCH Steering Group has overall responsibility for the project and reports to the Steering Group for the Coalition for Research to Improve Aboriginal Health which in turn reports to the Boards of the Sax Institute and the Aboriginal Health and Medical Research Council.

A Memorandum of Understanding was developed between each ACCHO and the study team. The Board of each ACCHO was asked to formally agree to participate in the study after having time to consider the Memorandum of Understanding, the full questionnaire and the study protocols.

### Data

**Figure 2:**
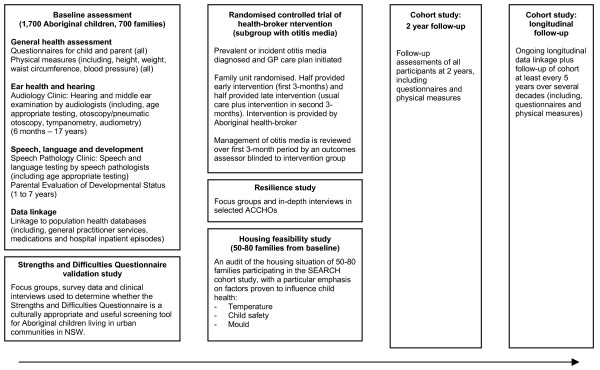
Overview of SEARCH data collection and intervention.

An overview of data collection in SEARCH is given in Figure 2.

### 1. Baseline questionnaire

The baseline questionnaire collects information on a range of demographic, social, lifestyle and health-related factors, including health service use, community and family resilience and quality and safety of housing.

An Aboriginal Research Officer interviews the person caring for children from participating families and obtains information using separate questionnaires for parents/caregivers and children aged 0-3 and 4-17 (see Table 1 for details). Participants aged 12-17 also fill in a self-completed questionnaire (Table 2).

**Table 1:** Data collected from parent/caregivers in the SEARCH baseline questionnaire, relating to their children and themselves.

	0-3 year old questionnaire	4-17 year old questionnaire	Parent/caregiver questionnaire
**Demographic and social factors**	Date of birth Relationship to parent/caregiver Duration of care Maternal substance use in pregnancy Childcare and preschool attendance	Date of birth Relationship to parent/caregiver Duration of care Maternal substance use in pregnancy Childcare and preschool attendance School attendance	Date of birth Aboriginality Education Income Forced separation or relocation of children Contact with the law Recent stressful life events

**Lifestyle factors**	Duration of breastfeeding Other dietary information	Duration of breastfeeding Other dietary information Physical activity	Physical activity Smoking Alcohol use Gambling

**General health and wellbeing**	Disease history Otitis media and hearing Injury	Disease history Otitis media and hearing Injury Oral health Mental health^1^ Speech and language	Disease history Mental health^2^ Resilience Wellbeing^3^

**Health service use**	Health service utilisation	Health service utilisation	Health service utilization Proximity to services

**Environment**			Housing quality Social capital Neighbourhood safety Walkability

^1 ^The Strengths and Difficulties Questionnaire: Scores range from 0-40, scores ≥ 17 are generally considered abnormal

^2 ^The Kessler 10 Psychological Distress Scale: Scores range from 10-50, scores ≥ 22 indicate high levels of distress

^3 ^The Personal Wellbeing Index: Scaled scores range from 0-100, the normative range for Australia is 73.4-76.4

**Table 2:** Self-reported questionnaire data collected at baseline from SEARCH participants aged 12 to 17 years.

	12-15 year old questionaire	16-17 year old questionaire
**Demographic and social factors**	Date of birth Relationship to parent/caregiver Schooling Contact with the law Cultural connectedness Discrimination	Date of birth Relationship to parent/caregiver Schooling Contact with the law Cultural connectedness Discrimination

**Lifestyle factors**	Dietary information Physical activity Smoking Alcohol use	Dietary information Physical activity Smoking Alcohol use Drug use Sexual activity

**General health and wellbeing**	Mental health^4^	Mental health^4^

^4 ^The Strengths and Difficulties Questionnaire: Scores range from 0-40, scores ≥ 17 are generally considered abnormal

Questionnaire content is based on the Western Australian Aboriginal Child Health Survey [4] and the New South Wales Child Health Survey [11], where possible.

### 2. Clinical measures

An Aboriginal Research Officer measures the height, weight, waist circumference and blood pressure of both parent/caregivers and children using the following methods:

#### Weight

Weight is measured in kilograms using Soehnle Electronic Infant Scales for infants and TCS 200 Electronic Body Scales for children and adults. Scales are set to zero and participants remove shoes, socks and outer heavy garments prior to measurement. Participants are instructed to stand on the centre of the scales without support and with their weight distributed evenly.

#### Height

The recumbent length of infants is measured using a SECA Medical Scale. The height of children (2 years and over) and adults is obtained using Harpenden Portable Stadiometers. Participants remove their shoes and socks and are instructed to stand up straight with heels firmly on the floor and shoulders relaxed while looking straight ahead. Height is measured to the nearest 0.1 cm at the end of a deep inward breath whilst the data collector applies gentle pressure to the area at the back of the neck, just underneath the skull.

#### Waist circumference

Waist circumference is measured to the nearest 0.1 cm at the end of normal expiration with the arms relaxed at the sides, using the cross hand technique. The measurement is taken at the narrowest point between the lower costal border and the iliac crest.

#### Blood pressure

Blood pressure is measured using an automated blood pressure monitor (Omron AIA) on the right arm, in an air-conditioned environment. Participants are encouraged to sit quietly for 5 minutes before measurements. Three readings are taken and the mean used in analysis. Different cuff sizes (child (17-22 cm), small adult (22-32 cm) and adult (32-42 cm)) are used depending on the mid arm circumference: analyses will be adjusted for cuff size.

### 3. Audiology and otoscopic assessment

All children aged 6 months or older undergo formal standardised, age-appropriate hearing assessment and also have tympanometry and otoscopy, as follows:

#### Audiometry

A trained audiologist conducts a formal hearing assessment, accounting for ambient noise measured by a Sound Level Meter set to dBA. Where the reading is over 45 dBA (poor test conditions) children are moved to another location for testing if possible. Children who are able to comply have pure tone audiometry to determine the average hearing thresholds (in decibels) across the frequency range 0.5 to 4.0 kHz for their best ear. Infants undergo brainstem evoked responses.

#### Tympanometry

Tympanometry determines the presence of fluid in the middle ear. Children are positioned appropriately with their heads held still. If discharge or wax is obscuring the ear it is cleaned with a tissue spear prior to examination. Tympanometry is performed using an Earscan Tympanometer and Audiometer on the left and then the right ear. Three measures are taken and the mean recorded.

#### Video otoscopy/pneumatic otoscopy

Children's ears are visually examined using the otoscope probe of the Inline Systems Flexiscope. A photograph is taken of each child's left and right ear. The video recorder is activated whilst pneumatic otoscopy is performed. A jet of air is squeezed into the ear canal and the response of the pneumatic membrane noted. This is repeated two to three times for each ear. The photos and video recordings are sent to participating Ear, Nose and Throat surgeons for their diagnoses.

### 4. Speech and development assessment

Children aged 1-7 years are assessed by a study speech pathologist. Age-standardized speech pathology tests are conducted assessing receptive and expressive language, speech, narrative and phonological awareness (see Table 3 for further details). The Parent Evaluation of Developmental Status questionnaire is also administered.

**Table 3:** Speech and language data gathered on SEARCH participants aged 1-7 years.

	Chronological Age			
	
Communication Domain	1 to 1 yr, 11 mths	2 to 2 yrs, 11 mths	3 to 4 yrs, 11 mths	5 to 6 yrs, 11 mths
**Receptive language**	CSBS	Reynell Test of Language Development - Revised	PLS-4	CELF-Australian (appropriate subtests)
**Expressive language**	CSBS	Language Sample	PLS-4	CELF-Australian (appropriate subtests)
**Speech**	CSBS	CSBS or PLS-4	DEAP Screening Assessment	DEAP Screening Assessment
**Narrative**	Not age appropriate	Not age appropriate	Narrative Story retelling Personal Narrative	Narrative Story retelling Personal Narrative
**Phonological Awareness**	Not age appropriate	Not age appropriate	PIPA	PIPA
				

CSBS Communication Symbolic Behaviour Scales. It assesses pre-linguistic language skills

CELF Clinical Evaluation of Language Fundamentals

DEAP Diagnostic Evaluation of Articulation and Phonology

PIPA Preschool and Primary Inventory of Phonological Awareness

PLS-4 Pre-school Language Scale Australian Adaptation

### Provision of individual results and clinical care of those requiring it

Parent/caregivers are provided with written and verbal feedback regarding all health information collected. Copies are also provided to the general practitioners (GPs) of consenting families. A case discussion among ACCHO GPs, paediatricians and speech pathologists is conducted for all children that undergo the Parent Evaluation of Developmental Status, hearing and speech pathology assessments. Where problems are identified, children are referred to their ACCHO GP and then to an appropriate specialist treatment provider,

### Follow-up and data linkage

SEARCH participants will have their health followed long term through repeat data collection, funding permitting, and through linkage of SEARCH data to routinely collected data on health and health services use, including hospitalizations, emergency department presentations and deaths. Funding is being sought for follow up of the cohort of 1700 participants (Wave 1), described here, at two (Wave 2) and four years after baseline (Wave 3), and recruitment of an additional 800 participants at the point of the two year baseline follow up, as part of Wave 2.

### Statistical methods and sample size

Descriptive statistics such as means, standard deviations (or medians and quartiles), numbers and percentages will be obtained for measures at each time point. Prevalences of conditions of interest with 95% confidence intervals will be reported; changes from baseline will be presented as differences in means or proportions (for example the distribution of those who newly develop conditions, improve or remain the same) with 95% confidence intervals. Hierarchical models (linear or logistic as appropriate for outcomes) will be used to examine relationships between explanatory variables and outcomes over time. Estimates will be adjusted for clustering of participants within ACCHOs and, where relevant, children within families. This is expected to be minimal, given the large catchment of each ACCHO and we have allowed an impact of a design effect of 1.2. We assume a significance level of 5% and power of 80% throughout the sample size calculations.

At baseline (Wave 1), a sample size of 1700 children allows for estimates of the prevalence of various conditions with precision of ±2.5% (for all ages) and ±4% (for 1-7 year olds) for binary outcomes and ±0.05 standard deviations (for all ages) to ±0.08 standard deviations (for 1-7 year olds) for continuous outcomes. If 2,500 children are recruited by the end of Wave 2, assuming that 80% of children will be followed up at each time point, 70% will have data at all three time points, and allowing for a design effect of 1.2, there will be an effective sample size of 2,080 children with baseline data, 1,660 with data at two time points (baseline and two or four year follow up), and 990 with data at all three time points (Wave 1 individuals only). This number will allow: estimation of prevalence of conditions with precision of ±2.1% (for all ages) to ±3% (for 1-7 year olds) for binary outcomes and ±0.04 standard deviations (for all ages) to ±0.08 standard deviations (for 1-7 year olds) for continuous outcomes; for detection of changes over time of 5% to 10% for binary measures and 0.1 to 0.2 standard deviations for continuous measures; for examination of factors relating to changes in health over time, there will be power to detect associations between explanatory variables and change over time of 10% to 15% for binary variables and 0.2 to 0.3 standard deviations for continuous measures.

### Preliminary characteristics of study population

Recruitment into SEARCH began in November 2008. As at end March 2010, recruitment was progressing at all four centres and 1010 children had joined the study. From those 446 children with complete data entry, participating children ranged in age from 2 weeks - 17 years old, with 144 aged 0-3 years, 147 aged 4-7 years, 75 aged 8-10 years and 79 aged 11-17 years (Table 4). Overall, 55% were male and 45% female. We anticipate completion of recruitment of 1700 children by the end of 2010.

**Table 4:** Characteristics of SEARCH participants with data entry completed up to September 2009.

CHILDREN	Males		Females		Total
	%	(n)	%	(n)	n
**Total**	55%	(247)	45%	(199)	446
**Recruitment site**					
Western Sydney	38%	(93)	34%	(67)	160
Tharawal	42%	(103)	43%	(85)	188
Awabakal	11%	(27)	15%	(30)	57
Riverina	10%	(24)	9%	(17)	41
**Age**					
0-3 years	31%	(77)	34%	(67)	144
4-7 years	33%	(82)	33%	(65)	147
8-10 years	18%	(44)	16%	(31)	75
11-13 years	12%	(30)	11%	(21)	51
14-17 years	6%	(14)	7%	(14)	28

**PARENTS/CAREGIVERS**	**Males**		**Females**		**Total**
	%	(n)	%	(n)	

**Total**	11%	(22)	89%	(176)	198
**Recruitment site**					
Western Sydney	41%	(9)	34%	(60)	69
Tharawal	36%	(8)	46%	(81)	89
Awabakal	14%	(3)	12%	(21)	24
Riverina	9%	(2)	8%	(14)	16
**Age**					
<20 years	0%	(0)	3%	(5)	5
20-29 years	41%	(9)	30%	(53)	62
30-39 years	18%	(4)	45%	(79)	83
40-49 years	18%	(4)	13%	(22)	26
≥50 years	23%	(5)	9%	(16)	21

Numbers do not always sum to total due to missing values

## Discussion

SEARCH is built on strong community partnerships, under Aboriginal leadership, and addresses community priorities relating to a number of under-researched areas. Currently, there are few longitudinal or intervention data available relating to Aboriginal health and little previous investigation of speech and language, mental health or links between environmental factors and health in this context. The Study represents a long term investment in Aboriginal Health research and will also build capacity through the training of more than ten Aboriginal researchers.

Like most cohort studies, SEARCH does not provide a representative sample of the Aboriginal population. However, this does not undermine its suitability for research based on internal comparisons within the cohort and longitudinal analyses. The conduct of Aboriginal health research is not straightforward [12] and a number of practical difficulties have been encountered in the course of the study, including identifying and retaining appropriate staff and identifying suitable premises. Importantly, most of the Study measures have not been validated for use in an Aboriginal sample - this is a pervasive problem in Aboriginal health research. We are addressing this issue by conducting validation work within the study (e.g. validation of the Strengths and Difficulties Questionnaire).

The primary SEARCH study will provide important information in itself. Furthermore, the recruitment, assessment and long term follow up, including linkage to routinely collected state and national population health data sets, of 1700 Aboriginal children creates a foundation for a wide variety of research to inform and influence interventions and service delivery. Projects to date that have been generated from the foundation of the SEARCH study include:

• A controlled trial to examine whether a community appointed health broker, who works directly with families, can improve the treatment of otitis media in Aboriginal children.

• A descriptive study investigating the housing needs of 70 Aboriginal families living in urban communities in NSW based around four issues: safety; insulation; mould; and vermin. Based on the information from the descriptive study, this project will then seek funding for a larger scale intervention targeting the issues of greatest need.

• A study of the validity of the Strengths and Difficulties Questionnaire (a screening tool for emotional and behavioural problems) to determine whether this instrument is a culturally appropriate and useful screening tool for Aboriginal children living in urban communities in NSW.

• A pilot study of an Aboriginal community designed and led early intervention program for young people suffering from depression or anxiety. This program involves participating in a range of activities designed to create positive peer relationships, increase pride in and knowledge of Aboriginal culture, build skills and learn about a range of health and social issues in an informal setting.

• A study using individual-level data from the SEARCH study to determine the type, quality and amount of green space available to participating Aboriginal families to: (i) describe patterns of physical activity, overweight and obesity; (ii) examine the self reported use of local green space for physical activity; (iii) explore barriers to the use of green space for exercise; (iv) Examine the relationship between green space, physical activity, overweight and obesity.

It is expected that other projects will be built on this foundation as SEARCH continues, particularly if resources are secured to conduct long term follow up.

SEARCH is on track to complete recruitment of 1700 urban Aboriginal children by late 2010. The study will provide unique long-term resource to investigate the causes and trajectories of health and illness in urban Aboriginal children and to identify potential targets for interventions to improve health.

## Competing interests

The authors declare that they have no competing interests.

## Authors' contributions

AW contributed to study design and conduct and acquisition of data, interpreted analyses and drafted the initial manuscript. EB contributed to study conception and design, interpreted analyses and drafted the initial manuscript. SR conceived of the study and participated in its design, conduct and coordination. JC conceived of the study and participated in its design and conduct. AC contributed to study conception, design and conduct. DF contributed to study conduct, coordination and community engagement. SE conceived of the study and participated in its design, conduct, coordination and community engagement. SB conceived of the study and participated in its design, conduct, coordination and community engagement. All authors were involved in revising the manuscript critically for important intellectual content and have given final approval of the version to be published.

## Pre-publication history

The pre-publication history for this paper can be accessed here:

http://www.biomedcentral.com/1471-2458/10/287/prepub
